# Robust associations between the 20-item prosopagnosia index and the Cambridge Face Memory Test in the general population

**DOI:** 10.1098/rsos.160923

**Published:** 2017-03-01

**Authors:** Katie L. H. Gray, Geoffrey Bird, Richard Cook

**Affiliations:** 1 School of Psychology and Clinical Language Sciences, University of Reading, Reading, UK; 2 Experimental Psychology Department, University of Oxford, Oxford, UK; 3 MRC Social, Genetic and Developmental Psychiatry Centre, Institute of Psychiatry, Psychology, and Neuroscience, King's College London, London, UK; 4 Department of Psychology, City, University of London, London, UK

**Keywords:** developmental prosopagnosia, PI20, self-report evidence

## Abstract

Developmental prosopagnosia (DP) is a neurodevelopmental condition, characterized by lifelong face recognition deficits. Leading research groups diagnose the condition using complementary computer-based tasks and self-report measures. In an attempt to standardize the reporting of self-report evidence, we recently developed the 20-item prosopagnosia index (PI20), a short questionnaire measure of prosopagnosic traits suitable for screening adult samples for DP. Strong correlations between scores on the PI20 and performance on the Cambridge Face Memory Test (CFMT) appeared to confirm that individuals possess sufficient insight into their face recognition ability to complete a self-report measure of prosopagnosic traits. However, the extent to which people have insight into their face recognition abilities remains contentious. A lingering concern is that feedback from formal testing, received prior to administration of the PI20, may have augmented the self-insight of some respondents in the original validation study. To determine whether the significant correlation with the CFMT was an artefact of previously delivered feedback, we sought to replicate the validation study in individuals with no history of formal testing. We report highly significant correlations in two independent samples drawn from the general population, confirming: (i) that a significant relationship exists between PI20 scores and performance on the CFMT, and (ii) that this is not dependent on the inclusion of individuals who have previously received feedback. These findings support the view that people have sufficient insight into their face recognition abilities to complete a self-report measure of prosopagnosic traits.

## 1. Introduction

Developmental prosopagnosia (DP) is a neurodevelopmental condition, characterized by lifelong deficits in facial identity recognition, despite normal intelligence, typical low-level vision and no history of brain damage [1–4]. Individuals with DP typically use non-face cues including voice, gait and hairstyle to recognize others. Consequently, they often experience great difficulties when non-face cues are unavailable or changed, or when familiar people are encountered out of context. DP is known to be a heterogeneous condition; for example, some individuals appear to perceive facial expressions normally [5], whereas others exhibit problems with facial expression perception [6]. Similarly, some individuals with DP recognize objects normally [7,8], while others exhibit broader object recognition deficits [9,10]. DP can be a socially debilitating condition often associated with social isolation, depression and anxiety, and reduced employment opportunities [11,12].

DP is not listed in the Diagnostic and Statistical Manual of Mental Disorders (DSM-5) [13] and currently no formal diagnostic criteria exist. Leading research groups therefore diagnose DP through the accumulation of convergent diagnostic evidence. Computer-based tests of face recognition ability, including the Cambridge Face Memory Test (CFMT [14]) and the Cambridge Face Perception Test (CFPT [10]), form a key part of most diagnostic batteries. Many authors also report performance on famous face recognition tests (e.g. [7,15,16]). In addition to scores on computer-based tests, however, self-report measures provide a complementary source of diagnostic evidence. For example, research groups routinely conduct diagnostic interviews and administer questionnaire measures that enquire about the face recognition experience of potential DPs (e.g. [17]). Where objective computer-based measures and subjective self-report measures provide convergent evidence of impairment, researchers can be confident about diagnosis and classification [18].

Historically, different research groups have employed bespoke self-report procedures, hampering the description and comparison of self-report data. In an attempt to standardize the reporting of self-report evidence, the *troublewithfaces.org* team recently published the 20-item prosopagnosia index (PI20), a short questionnaire measure of prosopagnosic traits suitable for screening adult samples for DP [19]. Respondents indicate the extent to which 20 statements describe their face recognition abilities and experiences. Agreement is rated on a five-point scale yielding scores ranging from 20 to 100. Sample items include: *I often mistake people I have met before for strangers; Without hearing people's voices I struggle to recognize them; I sometimes find movies hard to follow because of difficulties recognizing characters.* Scores on the questionnaire have been shown to effectively distinguish previously classified DPs from typical observers falling within the normal range of abilities [6,19,20].

As part of the validation procedures, the original PI20 paper described a highly significant correlation (*r* = −0.68, *p* < 0.001) between PI20 scores and performance on the CFMT [14]; a leading standardized measure of face recognition ability, employing a three-alternative-forced-choice match-to-sample design (see Validation Study 4 [19]). These results helped to confirm a key premise underlying the logic of the PI20; that individuals have sufficient insight into their face recognition ability to complete a self-report measure of prosopagnosic traits. However, the validation studies included a number of previously diagnosed DPs in the sample (approx. 21%). Some of these known DPs have been involved in previous research and had therefore received feedback from formal testing prior to administration of the PI20 questionnaire. It is conceivable that this feedback may have augmented their self-insight and thereby influenced how they completed the scale.

The possibility that the highly significant correlation described in the original PI20 paper [19] is an artefact of previously delivered feedback (e.g. results from formal testing) casts doubt on the crucial self-insight premise on which the PI20 is predicated, and potentially undermines the value of the scale as an independent source of diagnostic evidence. Here, we present novel data addressing this concern. We confirm that a significant relationship exists between PI20 scores and performance on the CFMT, and that this is not dependent on the inclusion of individuals who have previously received feedback from formal testing. We focus on the relationship with the CFMT as this is widely regarded as the most telling source of diagnostic evidence; while high scores on the CFMT typically exclude a diagnosis, members of DP samples sometimes score within the normal range on the CFPT (e.g. [15]) and on famous face recognition tests (e.g. [16]).

## 2. Method and results

Data were collected from two independent samples. None of the participants had completed formal testing of their face recognition ability. Self-reported face recognition ability played no role in the recruitment or selection of participants. The first sample (*n* = 142) was collected at City, University of London, and comprised adults recruited from the local subject pool (*M*_age_ = 29.23, s.d._age_ = 11.91, 56 males). Individuals were paid a small honorarium in return for their participation. The second sample (*n* = 283) was collected by undergraduate students at the University of Reading (*M*_age_ = 26.64, s.d._age_ = 13.16, 106 males). All participants completed both the PI20 and the CFMT. The first sample completed the PI20 before the CFMT; the second sample completed the CFMT and then the PI20. Participants were debriefed and given feedback only once both tasks had been completed. Ethical clearance was granted by the local ethics committees. The study was conducted in line with the ethical guidelines laid down in the 6th (2008) Declaration of Helsinki. All participants gave their informed consent.

**Figure 1.**
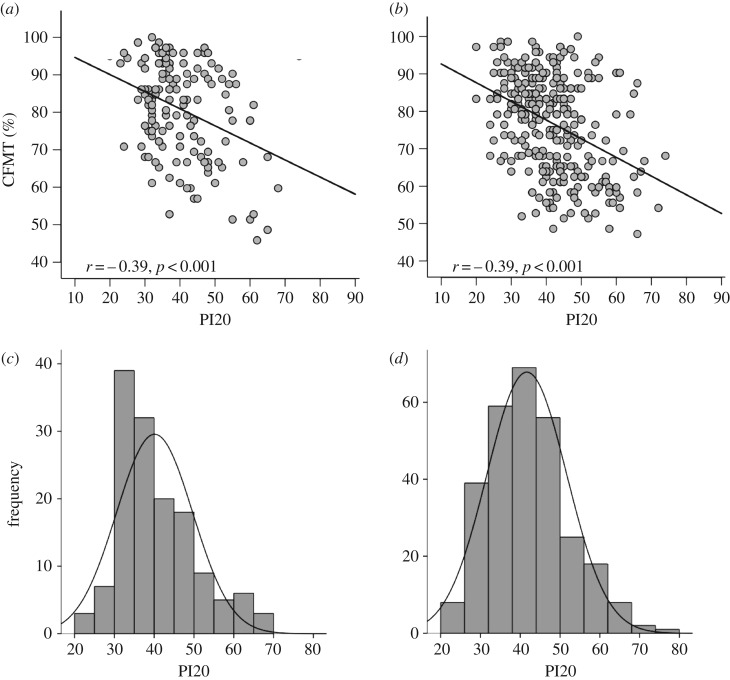
Associations between PI20 scores and performance on the CFMT for (*a*) the first sample collected at City, University of London (*n* = 142) and (*b*) the second sample collected through the University of Reading (*n* = 283). Both the first (*a*) and second (*b*) samples exhibited some indication of positive skewing. Note the frequency values differ between (*c*) and (*d*).

The first sample (*n* = 142) scored between 23 and 68 on the PI20 (*M* = 40.10; s.d. = 9.58) and between 45.8 and 100% on the CFMT (*M* = 80.65; s.d. = 12.79). The second sample (*n* = 283) scored between 20 and 74 on the PI20 (*M* = 41.70; s.d. = 10.10) and between 47.2 and 100% on the CFMT (*M* = 76.80; s.d. = 12.90). Three participants from the first sample, and nine from the second, yielded PI20 scores that exceeded the diagnostic cut-off (more than or equal to 65) suggested in the original study [19]. Crucially, we found highly significant correlations between participants' scores on the PI20 and CFMT in both the first sample, *r* = −0.394, *p* < 0.001 (figure 1*a*) and in the second sample, *r* = −0.390, *p* < 0.001 (figure 1*b*). The participants in these samples had no opportunity to use feedback from formal testing to inform their responses. These findings therefore lend further support to the view that people have sufficient insight into their face recognition abilities to complete a self-report measure of prosopagnosic traits.

## 3. Discussion

The correlations presented here represent important additions to the literature on the PI20 insofar as they estimate the relationship seen between PI20 scores and CFMT performance in the general population. Of the 110 observers who took part in the original validation study [19], 23 (21%) were known or suspected DPs. By contrast, the incidence of DP in the general population is thought to be approximately 2% [21,22]. A substantial number of DPs were included in the original sample in order to document the relationship between PI20 scores and performance on the CFMT across the entire range of abilities (i.e. normal and impaired), and thereby confirm the use of the PI20 as a diagnostic tool. Recently, the aim of the original study has been misunderstood; some authors have implied this correlation estimates the relationship between PI20 scores and performance on the CFMT in the general population [23–25]. However, this was *not* the aim of the original study; rather it sought to validate the PI20 as a diagnostic instrument [19]. It is very clear that the rate of incidence of DP in the general population is much lower than 21% [21,22].

As expected, the correlations observed in the present datasets (approx. −0.39) were weaker than those seen in the original validation study. Two factors are likely to contribute to this disparity. First, the range of abilities in the present samples is narrower than that employed in the original validation study. The variability within to-be-correlated variables will inevitably influence the strength of any correlation observed. By way of analogy, one may expect a weaker relationship between IQ and school achievement in samples of university students, than in samples drawn from the general population [26]. Consistent with this observation, a weaker correlation is also seen when the correlational analysis described in the original validation study (*n* = 110, *r* = −0.68) is restricted to those participants who did not describe face recognition problems (*n* = 87, *r* =−0.32). The strength of correlation seen in small samples drawn from the general population may be quite variable as it is influenced by the number of potential prosopagnosics identified. With larger samples, the correlation estimates are likely to stabilize.

Second, PI20 scores are ill-suited for estimating individual differences within the normal or superior range of abilities. Despite the correlations observed here, it is important to recognize that the PI20 is a measure of prosopagnosic traits, not a measure of face recognition ability *per se*. For example, observers in the 45th and 55th percentile of the general population will probably respond in very similar ways to items such as ‘*Anxiety about face recognition has led me to avoid certain social or professional situations.*’ Only people with very bad face recognition are likely to recognize such experiences; the rest of the population will not, irrespective of whether they have adequate, good or excellent face recognition. Unsurprisingly, PI20 scores from the typical population therefore exhibit some positive skewing (figure 1*c*,*d*), suggestive of asymmetric sensitivity. This feature is seen in several popular instruments used to screen for neurodevelopmental disorders (e.g. [27]).

Whether or not people have insight into their face recognition ability is a deceptively complex question; findings will probably depend on how estimates of self-reported ability are elicited and who is asked. Because the PI20 uses a number of concrete statements and easy-to-recognize anecdotes, respondents can interpret items even if they have had little cause to reflect on their ability. Estimating self-reported ability using abstract single-item measures (e.g. asking participants to rate their face recognition ability ‘compared with the average person’) may not be a fruitful approach [19]. Nevertheless, we note that self-report scores elicited using abstract one-shot measures *do* correlate significantly with objective measures of face recognition ability [24,25,28]. Individuals with extremely good or extremely bad face recognition ability (so-called ‘super-recognizers’ [29] and DPs, respectively) are also more likely to encounter situations in their daily lives which illustrate that face recognition is a distributed ability, and suggest where they might fall within that distribution. Unnuanced assertions that people lack insight into their ability (e.g. [18]) are therefore overly simplistic.

Cases of DP should not be diagnosed based solely on self-report evidence. However, when used properly, the PI20 provides independent diagnostic evidence that complements scores from objective computer-based tasks. There is a multitude of reasons why participants with typical face perception may score badly on computer-based tests, including boredom and fatigue, a lack of motivation, prioritization of response speed over accuracy, test anxiety, and manual and technical difficulties [19]. When tested on the CFMT, large undergraduate samples routinely yield numerous scores in the DP range [28]. However, in the absence of convergent self-report evidence, such scores should be treated with caution; the embarrassing social consequences of poor face recognition ensure that genuine sufferers are usually aware of their issue. The inclusion of self-report measures in diagnostic batteries also ensures that novel forms of DP do not go undetected. For example, difficulties perceiving dynamic faces, or problems learning faces from multiple encounters, will not be picked up by leading computer-based tests which assess perception of static unfamiliar faces only [10,14].

## Supplementary Material

Participants' scores on the CFMT and PI20

## Data accessibility

The datasets supporting this article are available as the electronic supplementary material.
